# Trace Detection of RDX, HMX and PETN Explosives Using a Fluorescence Spot Sensor

**DOI:** 10.1038/srep25015

**Published:** 2016-05-05

**Authors:** Chen Wang, Helin Huang, Benjamin R. Bunes, Na Wu, Miao Xu, Xiaomei Yang, Li Yu, Ling Zang

**Affiliations:** 1 Nano Institute of Utah and Department of Materials Science and Engineering, University of Utah, Salt Lake City, Utah 84112, USA; 2 Key Laboratory of Colloid and Interface Chemistry, Shandong University, Ministry of Education, Jinan 250100, PR China

## Abstract

1,3,5-trinitroperhydro-1,3,5-triazine (RDX), octahydro-1,3,5,7-tetranitro-1,3,5,7-tetrazocine (HMX), and pentaerythritol tetranitrate (PETN), the major components in plastic explosives, pose a significant threat to public safety. A quick, sensitive, and low-cost detection method for these non-volatile explosives is eagerly demanded. Here we present a fluo-spot approach, which can be employed for *in situ* detection of trace amount of explosives. The sensor molecule is a charge-transfer fluorophore, DCM, which is strongly fluorescent in its pristine state, but non-fluorescent after the quick reaction with NO_2_· (or NO_2_^+^) generated from the UV photolysis of RDX, HMX (or PETN). When fabricated within silica gel TLC plate, the fluo-spot sensor features high sensitivity owing to the large surface area and porous structure of the substrate. The sensor reaction mechanism was verified by various experimental characterizations, including chromatography, UV-Vis absorption and fluorescence spectroscopy, MS and ^1^H NMR spectrometry. The fluo-spot also demonstrated high selectivity towards RDX, HMX and PETN, as no significant fluorescence quenching was observed for other chemical compounds including common nitro-aromatic explosives and inorganic oxidative compounds. The DCM sensor can also be used as an economical spray kit to directly spot the explosives by naked eyes, implying great potential for quick, low-cost trace explosives detection.

Developing efficient detection methods for trace explosives has drawn intense attention due to increasing concerns about homeland security and public safety^1^. Compared to other commonly used explosives, the detection of 1,3,5-trinitroperhydro-1,3,5-triazine (RDX), octahydro-1,3,5,7-tetranitro-1,3,5,7-tetrazocine (HMX), and pentaerythritol tetranitrate (PETN) (Fig. 1a) remains difficult because of their low volatility and weak electron withdrawing ability^2^,^3^,^4^,^5^. Although numerous attempts to develop sensors for these explosives have been made, most sensors reported are limited to solution-based media or require long exposure time for vapor detection^6^,^7^,^8^. The lowest unoccupied molecular orbitals (LUMO) of these explosive compounds are located in high level, thereby requiring an even higher LUMO level of the sensor molecules to trigger the charge transfer process, which causes the fluorescence quenching in optical sensors or the conductivity change in chemiresistive sensors^9^. But even with this requirement fulfilled, the sensor materials’ instability and complicated synthesis of these sensor molecules may hinder the practical application^3^,^10^.

On the other hand, the decomposition of RDX and PETN was intensively studied under different circumstances^11^,^12^,^13^,^14^,^15^. The highly reactive intermediate species (e.g., radicals) produced from the decompositions can react quickly with the sensor molecule, providing a new detection pathway for RDX and PETN. Recently, it was found that RDX or PETN undergoes photolysis to generate the reactive intermediate species, NO_2_· radical or NO_2_^+^ cation, respectively, under the irradiation of UV light^13^. Both NO_2_· and NO_2_^+^ can be captured and detected by fluorescence sensor molecules. This decomposition mediated detection enables development of chemical sensors for RDX and PETN, as well as HMX (an analogue of RDX). To further improve the detection efficiency, and more importantly adapt the sensor mechanism into a solid detection system thus allowing for quick onsite screening, we herein design a fluorescence spot (fluo-spot) detection method with multiple strategies to optimize the sensor performance, as shown in Fig. 1c.

The fluorescence molecule used is 4-(dicyanomethylene)-2-methyl-6-(4-dimethylaminostyryl)-4H-pyran) (DCM), as shown in Fig. 1a. DCM is featured by its high fluorescence quantum yield (15% in solid state, measured over the DCM deposited on a polytetrafluoroethylene film) and unique electronic push-pull structure. The push-pull structure is a widely used design strategy for organic fluorescent molecules, as both the fluorescence efficiency and wavelength are very sensitive to the position and ability of the electron withdrawing or donating groups, which in turn can be changed by reacting with the analyte species^16^,^17^. In our work, the fluorescence of the DCM dye is completely quenched after its reaction with NO_2_· or NO_2_^+^ due to the destruction of the push-pull molecular structure. Combination of the high reactivity, strong initial fluorescence and the low cost based on the commercial availability makes DCM an ideal candidate for new sensor development. In order to optimize the sensor’s solid detection performance, a capillary is used to quantitatively load trace amount of DCM dye onto a thin-layer chromatography (TLC) silica gel plate to create a fluo-spot sensor. In this system, the DCM dye is dispersed into the porous silica substrate, which features a large surface area to ensure the efficient solid phase reaction with the target species. Upon weak UV irradiation from a hand-held UV lamp, ca. 1 pmol (~0.2 ng) of RDX, 1 pmol (~0.3 ng) of HMX or 1–10 pmol (~0.3 ng–3 ng) of PETN could be detected within 1 min using the DCM fluo-spot sensor by monitoring the fluorescence quenching. The sensor reaction mechanism shown in Fig. 1b was confirmed by the ^1^H NMR structural characterization of the final product after the photolysis reaction between the DCM and the explosives. To move the sensor into real application, unbiased detection of RDX was achieved on various common surfaces by using a solution spray technique.

## Results and Discussions

### Sensor characterization in solutions

**Figure 1:**
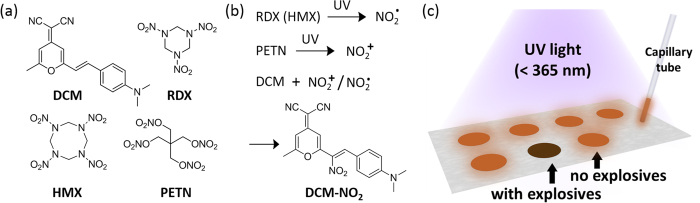
(**a**) Molecular structures of the fluorescence sensor, DCM, and the explosive compounds, RDX, HMX and PETN. (**b**) UV photolysis of RDX and PETN produces NO_2_· and NO_2_^+^, respectively; these reactive species can then be captured by DCM to produce the adduct DCM-NO_2_, leading to complete fluorescence quenching of DCM. (**c**) Scheme of the fluo-spot detection method.

**Figure 2:**
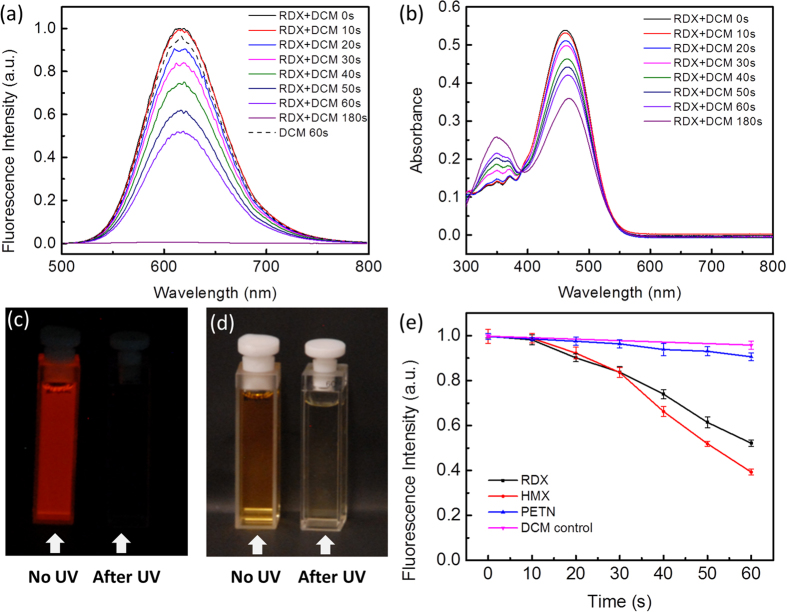
Fluorescence (**a**) and absorption (**b**) spectral change of DCM dissolved in acetonitrile in the presence of RDX before and after UV irradiation (254 nm) at varying time intervals. The dash black line in (a) shows the spectral change of the DCM solution but without RDX present after 60 s of UV irradiation; the slight decrease in intensity is due to photo-bleaching. (**c**) A fluorescence photograph showing the complete emission quenching of the DCM/RDX solution after 180 seconds of UV irradiation. (**d**) A bright field photograph taken for the same samples in (c), for which the dramatic color change indicates the conversion of DCM to DCM-NO_2_. (**e**) Decrease in fluorescence intensity of the acetonitrile solution of DCM in the absence and presence of the three explosives as a function of UV irradiation time. For all the experiments, the concentration of DCM is 1 × 10^−5^ mol·L^−1^, and the explosive compounds is 2 × 10^−5^ mol·L^−1^.

The sensor reaction of DCM was first characterized in molecular solution, as shown in Fig. 2. The three explosives used, RDX, HMX and PETN, are white crystalline powders and their acetonitrile solutions are transparent to visible light, absorbing mainly in the middle ultraviolet (MUV) region (Fig. S1a). So in this work, the 254 nm UV light was applied for the photolysis of these explosive compounds. The main absorption peak of DCM in acetonitrile solution is located around 460 nm and the fluorescence emission (in red color) is peaked at 620 nm (Fig. S1b,c). Due to the intermolecular self-quenching, the fluorescence intensity of a DCM solution does not increase linearly with the concentration of DCM, especially in higher concentration domain (Fig. S1d). To minimize this self-quenching effect, the concentration of DCM was kept at 1 × 10^−5^ mol·L^−1^ for the spectral characterization study. UV irradiation of the DCM solution containing RDX resulted in gradual decrease in fluorescence intensity of DCM, and complete fluorescence quenching was observed after 180 s of irradiation (Fig. 2a,c). This fluorescence quenching indicates the destruction of the charge transfer (push-pull) structure of DCM by reacting with the NO_2_· radical produced from photolysis of RDX. As parallel measured, significant absorption spectral change was also observed for the sample under the UV irradiation as shown in Fig. 2b, where the original charge transfer band centered at 460 nm decreased with an increase of the absorption band at 350 nm. This spectral change can also be seen from the dramatic color change of the DCM solution (Fig. 2d). An isosbestic point was obtained at ca. 390 nm in the absorption spectra, indicating stoichiometric conversion of DCM to DCM-NO_2_. These fluorescence and absorption spectral observations prove the sensor reaction mechanism shown in Fig. 1b. To further support the reaction pathway, a control experiment was performed by repeating the same spectral measurement but without addition of RDX. As a result, only slight decrease in fluorescence intensity was observed (Fig. 2a), apparently due to photo-bleaching (a common phenomenon of organic molecule). This clearly indicates that the significant spectral changes are related to the photolysis of RDX. Indeed, without the light irradiation, no significant fluorescence quenching could be obtained even in the presence of RDX.

The similar fluorescence quenching and absorption change were also observed for the other two explosives, HMX and PETN (Fig. S2). Control experiments also proved that the spectral change could only be observed under UV photolysis of HMX or PETN. The presence of the explosives, but without UV irradiation, did not lead to significant fluorescence quenching or absorption change. Figure 2e shows the decrease in fluorescence intensity of DCM as a function of UV irradiation time for the four samples, pure DCM solution and the ones with addition of RDX, HMX and PETN. It is clear that the fluorescence quenching caused by the three explosives is significantly larger than the photo-bleaching of DCM. Interestingly, the fluorescence quenching caused by PETN is not as significant as RDX or HMX. This could be due to the less efficient photolysis of PETN to produce the reactive species, NO_2_^+^. Indeed, PETN has less absorption for the 254 nm irradiation in comparison to RDX or HMX (Fig. S1a).

### Fluo-spot sensing in silica gel TLC plate

**Figure 3:**
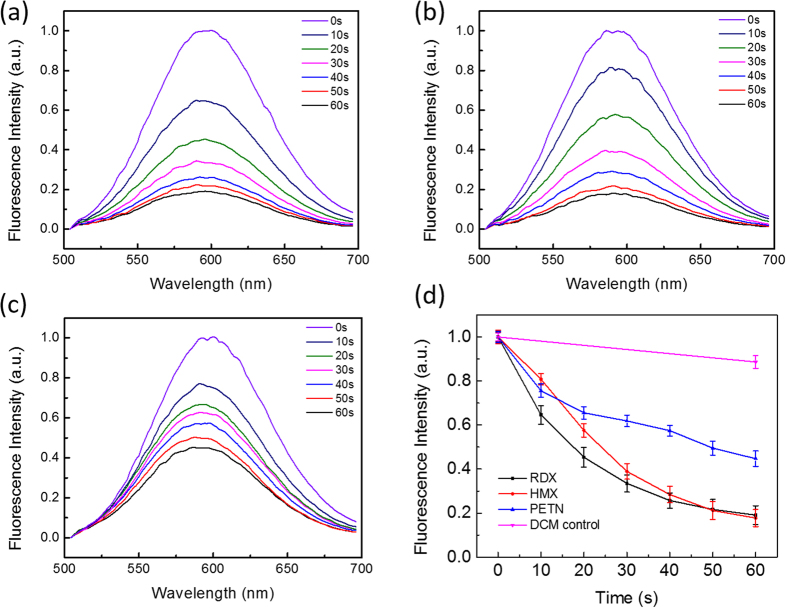
Fluorescence spectral change of DCM co-dispersed with (**a**) RDX, (**b**) HMX, and (**c**) PETN in silica gel TLC plate before and after UV irradiation (254 nm) at varying time intervals. (**d**) Decrease in fluorescence intensity of DCM as a function of UV irradiation time measured in the absence and presence of RDX, HMX and PETN. 10 pmol of DCM and 50 pmol of each of the explosives were used in all the experiments.

With the confirmed sensor reaction in solution phase, the DCM molecular system was adapted into solid matrix, to improve the practical application in trace explosives detection. To ensure the sufficient contact between DCM and the explosive compounds, the silica gel TLC plate was selected as the solid matrix because of its high porosity and large surface area^3^,^18^. A capillary was used to transfer the DCM and explosive solution onto the TLC plate to form a uniform round shape fluo-spot (as illustrated in Fig. 1c). This simple drop-casting method can quantitatively load the sensor molecules and potentially, enable the *in situ* detection on the surface of suspected objects. RDX, HMX and PETN were preloaded on the TLC plate in each spot, followed by loading of DCM at the same positions. After varying time intervals of UV irradiation, the fluorescence spectra of DCM were measured, as shown in Fig. 3a–c, for RDX, HMX, and PETN, respectively. Upon UV irradiation, the fluorescence intensity of DCM decreased gradually with the irradiation time, which is similarly to the sensor response observed in the solution of acetonitrile (Fig. 2e). Interestingly, the fluorescence peak of DCM measured in TLC plate was blue-shifted by ca. 20 nm in comparison to the fluorescence spectrum measured in acetonitrile solution. This fluorescence shift is a typical characteristic of a push-pull fluorophore (such as DCM), for which the charge transfer transition is very sensitive to the local dielectric property with the transition energy increasing with a decrease in dielectric constant (in the relative permittivity, from 37.5 of acetonitrile decreasing to 3.9 of silica)^19^.

Figure 3d shows the decrease of fluorescence intensity of DCM as a function of UV irradiation time measured in the presence of the three explosives. For comparison, the same quenching experiment was also measured for the DCM system without addition of explosive. Clearly, all the three explosives gave fast and significant fluorescence quenching, while the pure DCM showed only slight decrease in intensity (mostly due to the photo-bleaching). The quenching observed for RDX and HMX became faster in solid matrix of TLC plate compared to that in acetonitrile solution. For example, about 50% quenching was obtained after 20 s UV irradiation within TLC plate, while only 10% quenching was achieved in the solution phase. Such kinetics enhancement was found to be even more pronounced for PETN. In acetonitrile solution, 20 s of irradiation led to only a few percent fluorescence quenching, while within TLC plate about 30% quenching was obtained. This significant increase in quenching kinetics is mostly owing to the more concentrated molecular dispersion of DCM and explosives within the microporous silica gel matrix of the TLC plate, wherein the locally produced NO_2_· or NO_2_^+^ species will react quickly with DCM molecules in proximity. The highly localized reaction in solid state consumes the reactants faster in the initial stage, and as the amount of reactants decreases the reaction becomes slower. This is exactly the kinetics profile as shown in Fig. 3d. In contrast, the molecules in a solution are more sparsely diapered and the bi-molecular reaction is primarily limited by the diffusion. As a result, the reaction remains slow and follows approximately linear relation as shown in Fig. 2e. The fast initial quenching observed for the solid state is highly conducive to quick detection of explosives, ideally just within seconds.

**Figure 4:**
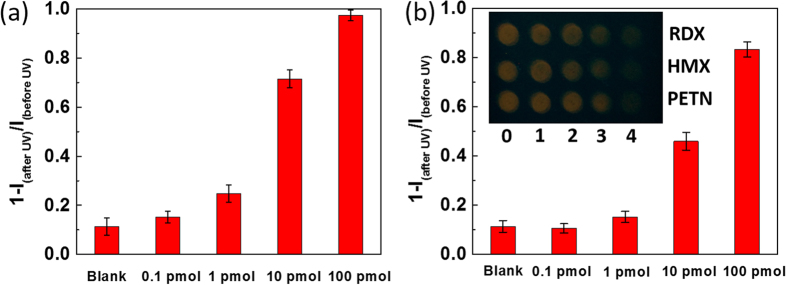
Fluorescence quenching yields of the fluo-spots of DCM containing different amounts of RDX (**a**) and PETN (**b**). Inset: A photograph showing increasing levels of fluorescence quenching of the three sets of fluo-spots upon increasing the amounts of RDX, HMX and PETN: 0 (no explosive), 1 (0.1 pmol), 2 (1 pmol), 3 (10 pmol), and 4 (100 pmol). The fluo-spots (each containing 10 pmol of DCM) were drop-cast on a silica TLC plate and exposed to the same intensity of 254 nm irradiation for 1 min.

The limit of detection (LOD) of the DCM sensor is primarily determined by the stoichiometric reaction between DCM and the reactive species, NO_2_· or NO_2_^+^, generated from UV photolysis of the explosives. On one hand, the smaller amount of DCM used, the lower the LOD would be assuming a complete stoichiometric reaction is achieved. However, on the other hand, too little DCM causes difficulty in measuring the fluorescence signal, *i.e.* diminished signal-to-noise. To balance this tradeoff, an optimal amount of DCM, 10 pmol, was used to fabricate the fluo-spots. By changing the amount of explosives (RDX, HMX and PETN) mixed with the DCM, different levels of fluorescence quenching were observed for the fluo-spots, enabling estimation of the LOD (Fig. 4). Briefly, varying amounts of the explosive (0.1–100 pmol) were preloaded on the TLC plate using capillary drop-casting method, forming a series of spots as shown in Fig. 4 inset. Then 1 μL of 1 × 10^−5^ mol·L^−1^ DCM solution was deposited using a capillary onto each of the spots. After drying under nitrogen flow, the fluo-spots thus fabricated were irradiated under fixed intensity of 254 nm light for 1 min. By measuring the fluorescent intensity of the fluo-spots containing different amounts of explosives (here taking RDX and PETN as the examples) in comparison to the intensity of the spot containing only DCM (a blank control), one can calculate the fluorescence quenching yield, 1-(I_after UV_/I_before UV_), depending on the amount of explosive added as shown in Fig. 4a,b. Due to the slight UV-bleaching of DCM, the 0.1 pmol samples show no significant difference to the blank spot because of the large overlap between their error bars. Based on this evaluation criterion, the LOD for RDX is determined as 1 pmol (or ~200 pg), and for PETN it should be in the range of 1–10 pmol (or 300 pg–3 ng). The same experiments were also performed for HMX, which demonstrated the same level of LOD as RDX. The sub-nanogram detection affords sufficient sensitivity for practical screening of explosives through surface particulate sampling (e.g., swabbing). It is commonly believed that a typical fingerprint may contain many explosives particles, with a total mass often on the order of 100 microgram^20^.

**Figure 5:**
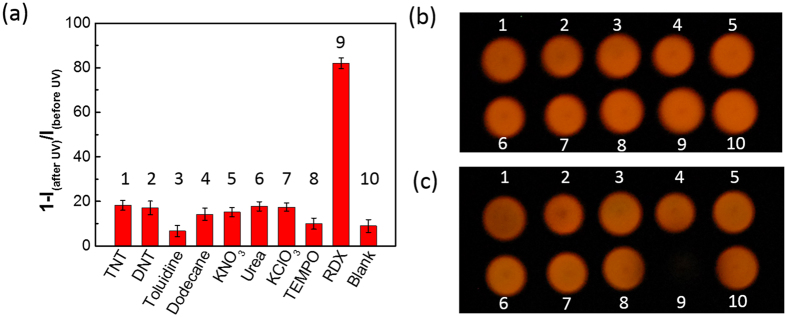
(**a**) Fluorescence quenching yields of the 10 fluo-spots of DCM containing different explosive compounds and other solid chemical interferents after 1 min 254 nm UV irradiation; the result of the blank sample (containing only DCM) is also shown for comparison. (**b**) A typical photograph showing the fluorescence emission of the 10 fluo-spots before UV irradiation. (**c**) A photograph taken for the same fluo-spots after 1 min UV irradiation. All the fluo-spots were deposited on the silica TLC plate, and each spot contains 0.1 nmol of DCM and 1 nmol of explosive or other chemical compounds as indicated in the plots.

Besides the sensitivity, the selectivity is another criterion to evaluate a sensor’s performance against potential interference. To evaluate the selectivity of the DCM fluo-spot sensor (i.e., only demonstrating response to three explosives RDX, HMX or PETN), eight chemical compounds were selected as the potential interferents to perform the same fluo-spot testing as described above. The testing results are shown in Fig. 5. All the eight interferents demonstrated about the same or slightly higher fluorescence quenching compared to the blank (DCM only) sample, which always exhibits some degree of quenching due to UV photo-bleaching. However, the presence of the same amount of RDX resulted in much higher fluorescence quenching, indicating the possibility of distinguishing RDX from those chemical interferents. The sensing selectivity thus observed for RDX is also applicable to HMX and PETN, considering their comparable fluorescence quenching capability as RDX (Fig. 3–4). The high selectivity attained for RDX, HMX and PETN is mainly owing to the unique sensor reaction pathway mediated by NO_2_· or NO_2_^+^ (Fig. 1b). Among the eight chemical interferents, 2,4,6-trinitrotoluene (TNT) and its analogue 2,4-dinitrotoluene (DNT) represent the common nitro-based explosives, and KNO_3_ and KClO_3_ are the oxidative components of inorganic explosives. The slightly increased quenching observed for these explosives (compared the blank sample) is likely due to the enhanced photobleaching (via photo-oxidation) facilitated by these oxidative compounds (functioning as electron acceptor). For the similar reason the slightly increased quenching observed for urea could be due to the photo-reduction caused by the electron donating of urea. Additionally, (2,2,6,6-Tetramethylpiperidin-1-yl)oxyl (TEMPO), a free radical species stable under ambient condition, was also selected as an interferent to repeat the same sensing test. As shown in Fig. 5b mixing DCM with TEMPO did not result in fluorescence quenching, indicating the fluorescence quenching of DCM observed above is not due to non-specific electron or energy transfer between DCM and radicals, but rather to the specific addition reaction between DCM and NO_2_· or NO_2_^+^. Such specific reaction (as to be confirmed below) enables the detection selectivity towards RDX, HMX and PETN. On the other hand, TEMPO is proven an effective capture for NO_2_· radical, and thus can be used as an additive in the fluo-spot sensor to help examine the sensor reaction pathway^21^. As shown in Fig. S3, about 78% fluorescence quenching was obtained for the fluo-spot sensor (containing 0.1 nmol of DCM and 1 nmol of RDX) after 1 min UV irradiation at 254 nm. In comparison, when 1 nmol of TEMPO was added into the fluo-spot, the fluorescence quenching efficiency was decreased to 53% by the same amount of RDX. Such decreased quenching supports the assumption that the observed fluo-spot sensing is mediated by the NO_2_· radical, which is produced by UV photolysis of RDX, and now is competitively consumed by TEMPO.

### Sensor reaction mechanism

To study the mechanism of the UV-induced fluorescence quenching of DCM by the explosives, we aimed to reveal the product of the reaction. It was reported that upon UV irradiation, PETN mainly releases nitrite cations (NO_2_^+^), while RDX or HMX releases nitrite radicals (NO_2_·)^13^. To verify whether RDX and PETN form the same product with DCM upon the UV irradiation, two DCM fluo-spots were fully reacted with excess RDX and PETN separately. Then both spots were run for TLC and demonstrated the same retention factor (R_f_) as shown in Fig. S4a. The same R_f_ value usually means the same product. To further confirm this, we introduced NO_2_BF_4_ salt to react with DCM in an acetonitrile solution, where the NO_2_^+^ cation dissociated from the salt would react with DCM to produce the product DCM-NO_2_, as indeed observed by the quick color fading of the solution. As shown in Fig. S4b, the visible absorption of DCM-NO_2_ in the region of 400–500 nm decreased about 50% compared to that of pristine DCM. This spectral change tendency is identical with what was observed for the fluo-spot of DCM mixed with RDX under UV irradiation (Fig. 2b). Also the same as observed for the fluo-spot, complete fluorescence quenching of DCM was also obtained after reacting with NO_2_BF_4_ in the solution. The reaction mixture of DCM and NO_2_BF_4_ salt was then examined for TLC along with the fluo-spots containing RDX and PETN. As expected, all the three samples run at about the same R_f_ value (Fig. S4a), again indicating the same product, DCM-NO_2_. As comparison, significantly different R_f_ was obtained for the pristine DCM run on the same TLC.

**Figure 6:**
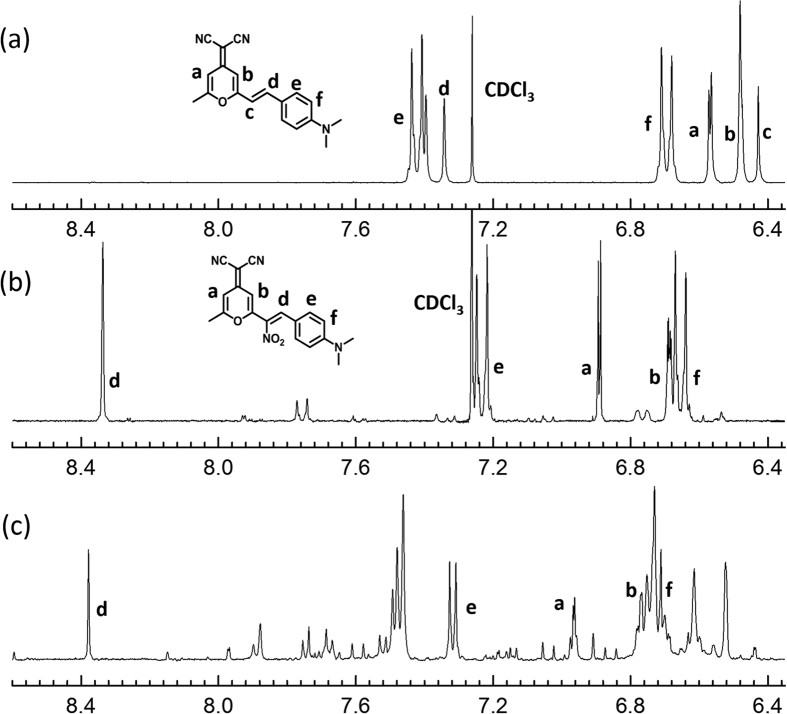
Partial ^1^H NMR spectra of (**a**) DCM and (**b**) DCM-NO_2_ in CDCl_3_, and (**c**) the unpurified product from the reaction of DCM and RDX in an acetonitrile solution after 2 min of 254 nm irradiation. The letters “a” to “f” mark the different hydrogen sites that are assigned to the chemical shifts in ^1^H NMR spectra.

To characterize the molecular structure of the product of DCM and NO_2_BF_4_ salt, the reaction mixture was purified by running through a silica gel chromatography column and the purified compound was subjected to mass spectrum (MS) analysis, showing a mass of 348.12, matching the structure of DCM-NO_2_ (Fig. S5). The mono-substitution of NO_2_ may be positioned at any hydrogen site of the conjugated styrene part, which is electron rich and renders the attack by electron deficient species like NO_2_· and NO_2_^+^ 22. Calculation of the DCM molecule based on density-functional theory reveals the charge distribution among the eight carbons within the styrene part (Fig. S6), in which the ethylene carbon connected to the pyran moiety possesses the most negative charge density. We suspected that this would be the position for nitro group (−NO_2_) substitution, leading to the product of DCM-NO_2_ in the structure shown in Fig. 1b. To finally verify this proposed structure, ^1^H NMR spectra were measured for DCM and DCM-NO_2_ purified from the reaction between DCM and NO_2_BF_4_, and part of the spectra are shown in Fig. 6a,b, respectively. For DCM molecule, all the peaks shown can be assigned to the six hydrogens (marked “a” to “f”) connected to the central conjugation backbone according to the chemical shifts and coupling constant (J) values. Upon converting to DCM-NO_2_, the peak corresponding to “c” hydrogen disappears, indicating the substitution of −NO_2_. Meanwhile the neighbor hydrogen at “d” undergoes a large chemical shift change from 7.34 to 8.34 ppm, due to the electron withdrawing substitution at “c”. The J values of the hydrogens at “a”, “b”, “e” and “f” sites remain unchanged (J_ab_ = 2.1 Hz, J_ef_ = 9.0 Hz, despite slight change in chemical shifts), implying that these positions are intact during the reaction. These ^1^H NMR results confirm the mono-substitution at the “c” position, consistent with the molecular weight measured by MS. Moreover, the large chemical shift of “d” peak can be used as a signature of the −NO_2_ substitution reaction, which can be monitored during the UV-photolysis sensing of RDX or PETN. Indeed, as shown in Fig. 6c, the ^1^H NMR spectrum measured over the raw product from the UV-photolysis of DCM and RDX exhibits the same “d” peak located at 8.38. Also shown are the other two characteristic peaks at “a” and “f”, which have the J values, chemical shifts and ratio of integration all the same as the ones measured for the pure DCM-NO_2_ compound (Fig. 6b). This further confirms that the sensor reaction within the fluo-spot is through the mono-substitution by NO_2_· and NO_2_^+^.

### Potential application of fluo-spot sensor

An ideal fluorescence quenching based sensor should have high fluorescence emission in the pristine state and lowest emission in the product state. DCM satisfies these requirements with a high fluorescence quantum yield of 15% in solid state and almost zero emission at the DCM-NO_2_ state (quantum yield <0.1%, below the measurability of the instrument used). This large quenching ratio can also be seen from the comparison of the fluorescence spectra measured over the DCM and DCM-NO_2_ solutions in acetonitrile at the same concentration (Fig. S3c), where the fluorescence intensity is different by two orders of magnitude. The potential of using the fluo-spot sensor in detection of the non-volatile explosives like RDX, HMX and PETN has already proven in the observations shown in Figs 3, 4, 5, which demonstrate both the high sensitivity and selectivity. To further prove the potential in practical applications, where the substrate could be any materials and the location of explosives is usually unknown, we adapted the fluo-spot sensor into a direct spray detection kit as shown in Fig. S7a. Two common substrates, a package cardboard and a dollar bill, were selected, onto which about 100 μg RDX was pre-loaded in different patterns (just for vision purpose in this experiment). Then a solution of DCM (3 × 10^−3^ mol·L^−1^ in a 1:1 vol acetonitrile:ethanol solvent) was sprayed onto the surface. After exposure to 254 nm irradiation for 2 min, the presence of RDX was clearly revealed as dark (quenched) area in contrast to the bright uniform fluorescence surrounding. These results demonstrate the feasibility of detecting RDX (HMX, PETN as well) on the common substrates even with naked eyes. Moreover, the DCM sensor kit is also featured by its low cost, which is always an issue when considering the real world usage of explosive sensors. DCM can be prepared easily under $100 per gram, and 1 g of DCM can make 330 million fluo-spots, or spray over 55 m^2^ on the surface. Combination of these facts will facilitate the practical application of the DCM sensor developed in this study.

## Conclusions

In this work, we demonstrate the fluo-spot approach for the trace detection of RDX, HMX and PETN. The fluo-spot sensor is based on a strongly fluorescent molecule, DCM, which converts into a non-fluorescent molecule, DCM-NO_2_, by reacting with NO_2_· or NO_2_^+^ that can be generated from the UV photolysis of RDX (HMX) or PETN, respectively. Such radical reaction mediated fluorescence quenching was proven fast and highly efficient in both solution and solid states. The reaction mechanism has been characterized and confirmed by systematic experimental methods including chromatography, UV-vis absorption and fluorescence spectroscopy, MS and ^1^H NMR spectrometry. When fabricated within silica gel TLC plate, the fluo-spot sensor exhibited high sensitivity owing to the large surface area and porous structure of the TLC substrate; the LOD was determined as low as 0.2 (0.3) ng and 0.3–3 ng for RDX (HMX) and PETN, respectively. The fluo-spot also demonstrated high selectivity towards the three explosives, as no significant fluorescence quenching was observed for other chemical compounds including the nitro-explosives and inorganic explosives. The DCM sensor can also be adapted into a simple spray kit to directly reveal the presence of explosives by naked eyes, providing great potential for quick, low cost trace explosives detection.

## Methods

### Materials and synthesis of DCM-NO_2_

RDX, HMX, PETN, DNT and TNT were obtained from Dyno Nobel. DCM and other compounds were purchased from Sigma-Aldrich (>98% purity) and used without further purification. The TLC plate (5554/7, silica gel 60 F_254_) was purchased from EMD Chemicals Inc. Compound DCM-NO_2_ was synthesized by reacting DCM with NO_2_BF_4_ salt. Briefly, 50 mL of 1 mM DCM in dry acetonitrile was mixed thoroughly with 50 mL of 1 mM NO_2_BF_4_ also in dry acetonitrile at 0 °C. After 10 min, KOH dissolved in dry acetonitrile was added to the reaction mixture to adjust the pH to be neutral. The mixture was then dried with a vacuum rotary evaporator. The raw product thus obtained was separated and purified by running through a silica gel chromatography column using 1:1 (v/v) hexane and ethyl acetate as eluent. The DCM-NO_2_ compound obtained was dried in a vacuum oven at room temperature, giving a yellow powder (yield of ca. 26%). ^1^H NMR (300 MHz, CDCl_3_): *δ* = 8.34 (s, 1H), 7.25–7.22 (d, *J* = 9 Hz, 2H), 6.89 (d, *J* = 2.1 Hz, 1H), 6.69 (d, *J* = 2.1 Hz, 1H), 6.67–6.64 (d, *J* = 9 Hz, 2H), 3.12–3.10 (m, 6H), 2.40 (d, *J* = 0.6 Hz, 3H). MS (ESI): *m/z* calculated: 348.12; found: 349.13 [M + H]^+^.

### Spectral characterization and UV irradiation

Solution samples of DCM and the explosives were prepared by dissolving the compound powders into dry acetonitrile, for which different concentrations were made. The absorption spectra were measured on an Agilent Cary 100 series UV-Vis spectrophotometer using the 6Q grade quartz cuvette, and the fluorescence spectra were measured on an Agilent Cary Eclipse Fluorescence Spectrophotometer. The fluorescence quantum yields of DCM and DCM-NO_2_ in solid state were measured over the compounds deposited onto a polytetrafluoroethylene film using the integrating sphere method equipped with a Hamamatsu Absolute PL Quantum Yield Spectrometer (C11247). For the time-course spectra measurements under UV irradiation, the same solution containing DCM and the explosives were parallel distributed into seven quartz cuvettes and six of them were subject to UV irradiation, while the rest one was used as the non-irradiated control. The six samples were taken out after varying time intervals of irradiation and measured for the absorption and fluorescence spectra, which can be compared to the non-irradiated sample. The UV irradiation was performed with a handheld UV lamp (model: UVGL-58, 6 Watt, 254/365 nm, P/N 95-0007-05, Upland, CA, USA).

### Preparation of fluo-spot sample and fluorescence measurement

The fluo-spot samples were prepared using a capillary drop-casting method as illustrated in Fig. 1c. Stock solutions of DCM, the explosives and interferents were prepared in dry acetonitrile at different concentrations. A glass capillary (inner diameter ca. 0.5 mm) was used to absorb the stock solution with amount controlled at 5 mm in height, which gives the volume of solution taken to be about 1 × 10^−6^ L. Then the one micro-liter solution was transferred onto the TLC plate by gently contacting the capillary to the substrate, forming a spot with a diameter of about 5 mm. The molar amount of the compounds thus loaded can be adjusted by the solution concentration. The loaded TLC plate was then dried under a nitrogen gas flow for 1 min. Considering the fact that the spot prepared from the explosives is colorless, to assure the following up loading of DCM to be within the same spot area, the pre-deposited spot center was marked by a dot using a pencil. For quantitative fluorescence intensity measurement, the fluo-spots were prepared in an array following the pattern of a standard 96-well microplate, so that the whole TLC plate could be read under a microplate reader (here carried out with an Agilent Microplate Reader, model G9810A, attached to the Agilent Cary Eclipse Fluorescence Spectrophotometer). By measuring the fluorescence intensity before (I_before UV_) and after (I_after UV_) UV irradiation, one can calculate the quenching efficiency, 1 − (I_after UV_/I_before UV_).

## Additional Information

**How to cite this article**: Wang, C. *et al.* Trace Detection of RDX, HMX and PETN Explosives Using a Fluorescence Spot Sensor. *Sci. Rep.* **6**, 25015; doi: 10.1038/srep25015 (2016).

## Supplementary Material

Supplementary Information
