# Supplementary material for: Trace Detection of RDX, HMX and PETN Explosives Using a Fluorescence Spot Sensor
Source: Sci Rep. 2016 May 5;6:25015. doi: 10.1038/srep25015 (PMC4857100; doi:10.1038/srep25015)
Supplement: Supplementary Information [file srep25015-s1.pdf]

Supplementary Information for

Trace Detection of RDX, HMX and PETN Explosives Using a Fluorescence Spot Sensor

Chen Wang[1,*], Helin Huang[1,*], Benjamin R. Bunes[1], Na Wu[1], Miao Xu[1], Xiaomei Yang[1], Li Yu[2], and Ling Zang[1]

[1]Nano Institute of Utah and Department of Materials Science and Engineering, University of Utah, Salt Lake City, Utah 84112, USA
[2]Key Laboratory of Colloid and Interface Chemistry, Shandong University, Ministry of Education, Jinan 250100, PR China
[*]These authors contributed equally to this work.
Correspondence and requests for materials should be addressed to L.Z.
(email: lzang@eng.utah.edu)

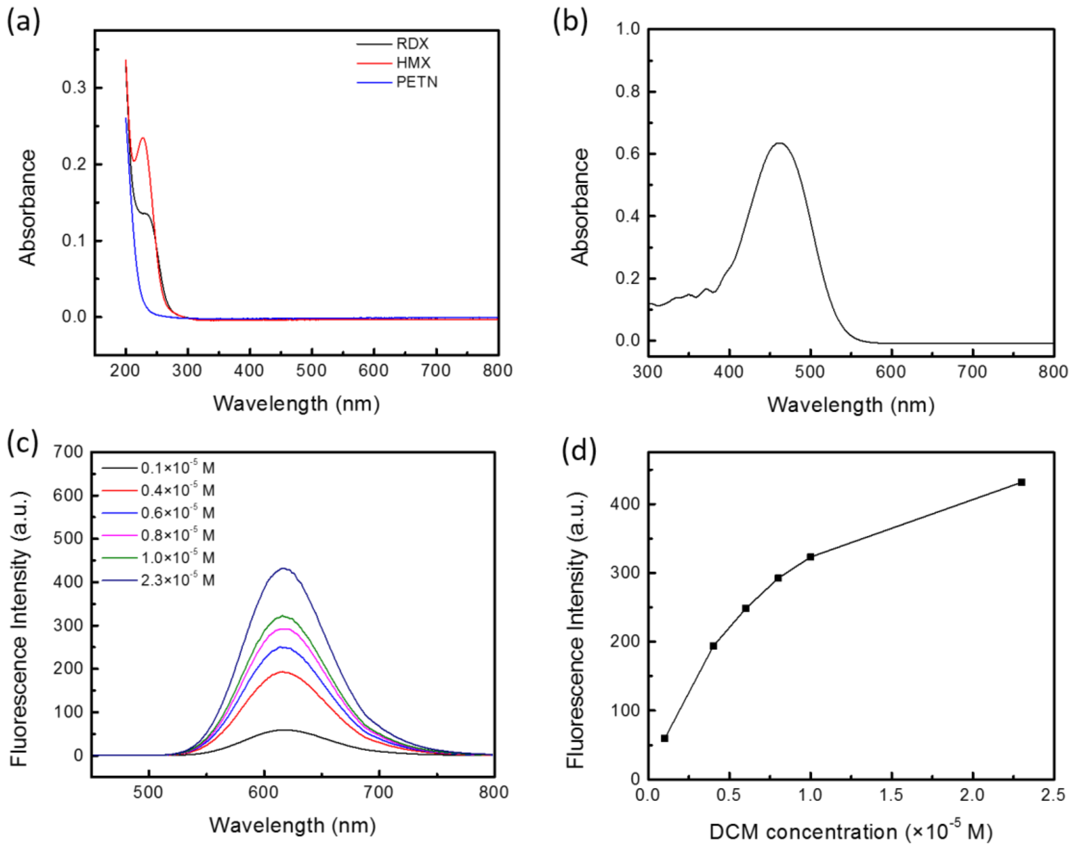

Figure S1. (**a**) The absorption spectra of RDX (black), HMX (red) and PETN (blue) in acetonitrile solutions, 1×10^{-5} mol·L^{-1}. (**b**) The absorption spectrum of DCM in an acetonitrile solution, 1×10^{-5} mol·L^{-1}. (**c**) The fluorescence spectra measured for the DCM solutions in acetonitrile at different concentrations. (**d**) The maximum fluorescence intensity as a function of the concentration of DCM solution.

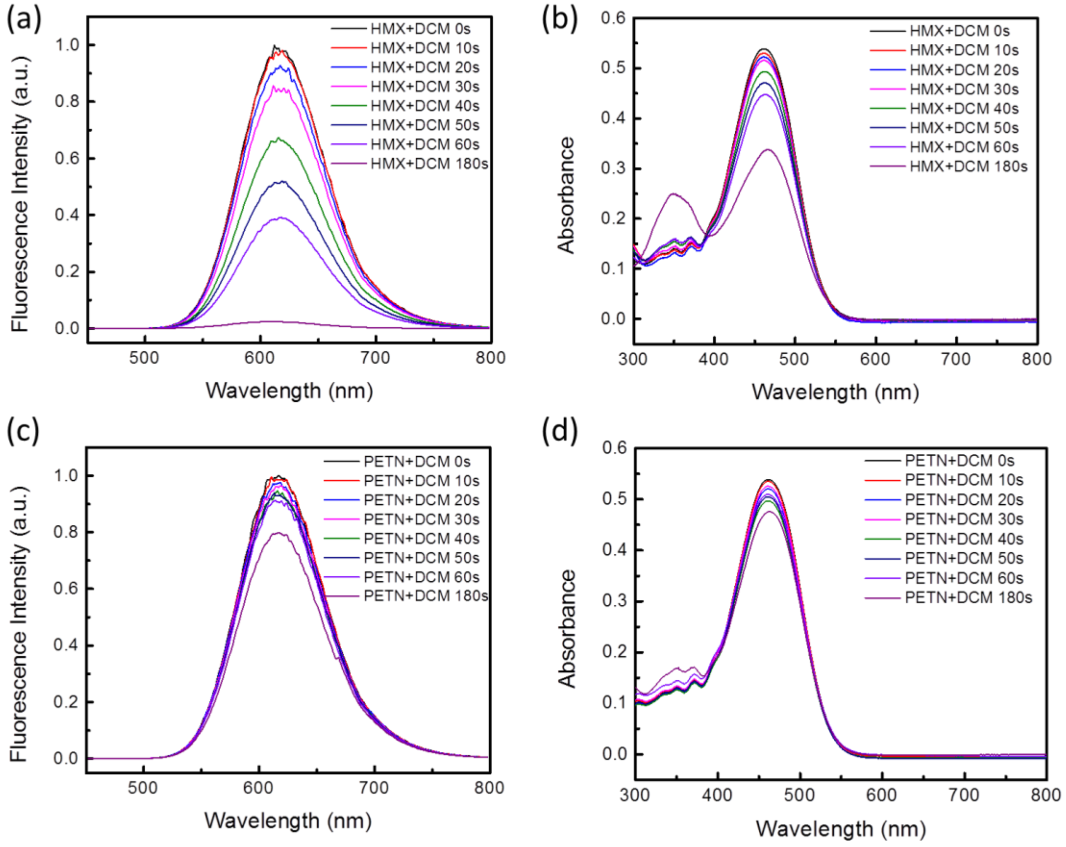

Figure S2. Fluorescence (**a**, **c**) and absorption (**b**, **d**) spectral change of DCM dissolved in acetonitrile in the presence of HMX (**a**, **b**) and PETN (**c**, **d**) before and after UV irradiation (254 nm) at varying time intervals. The concentration of DCM was 1×10^{-5} mol·L^{-1} and that of the explosives was 2×10^{-5} mol·L^{-1}.

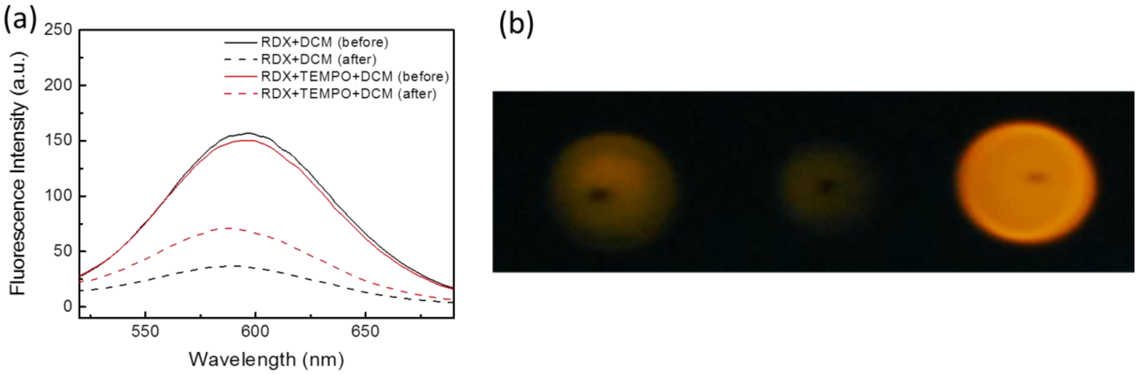

Figure S3. (**a**) Comparison of the fluorescence spectra measured over four samples: fluo-spot samples of DCM mixed with RDX before (solid black) and after (dash black) 1 min of UV irradiation at 254 nm; and fluo-spot samples of DCM mixed with RDX and TEMPO before (solid red) and after (dash red) the same irradiation. (**b**) the photograph taken for the fluo-spots (from left to right) of DCM with RDX and TEMPO, DCM with RDX, and DCM alone, all after 1 min of UV irradiation. Each fluo-spot contains 0.1 nmol of DCM; the left and middle spots also contain 1 nmol explosives; the left spot also contains 1 nmol TEMPO.

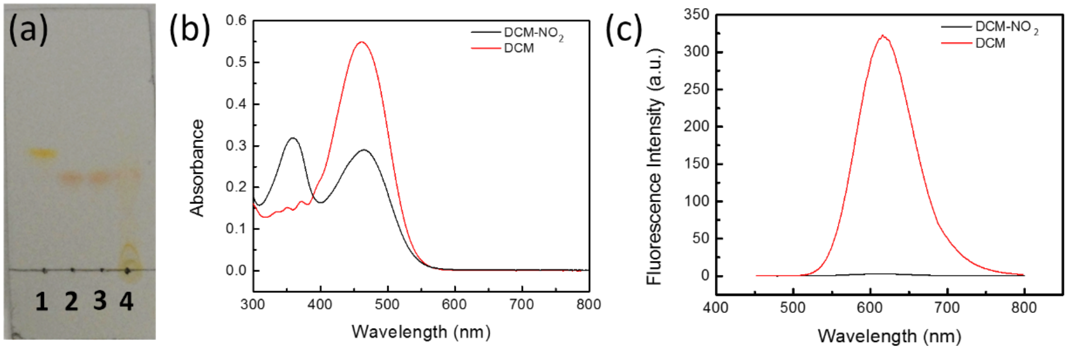

Figure S4. (**a**) The photograph taken on a silica gel TLC plate run with four samples: (1) 1 nmol of pure DCM, (2) 1 nmol DCM mixed with 5 nmol RDX after 3 min of 254 nm irradiation, (3) 1 nmol DCM mixed with 5 nmol PETN after 3 min of 254 nm irradiation, (4) the reaction mixture of DCM and NO_2BF_4 at 0 ℃ in acetonitrile (both at 1×10^{-3} mol·L^{-1}). The eluent solvent was 1:1 (v/v) hexane and ethyl acetate. (**b**) The absorption and (**c**) fluorescence spectra of DCM (red) and DCM-NO_2 (black) in acetonitrile solution (both at 1×10^{-5} mol·L^{-1}).

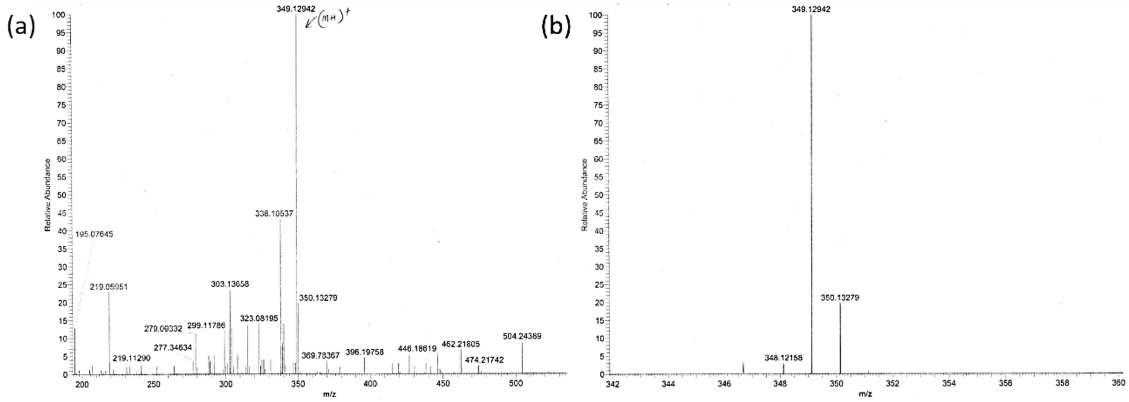

Figure S5. (**a**) The mass spectrum obtained for DCM-NO_2. (**b**) The zoomed-in view of the mass region of DCM-NO_2.

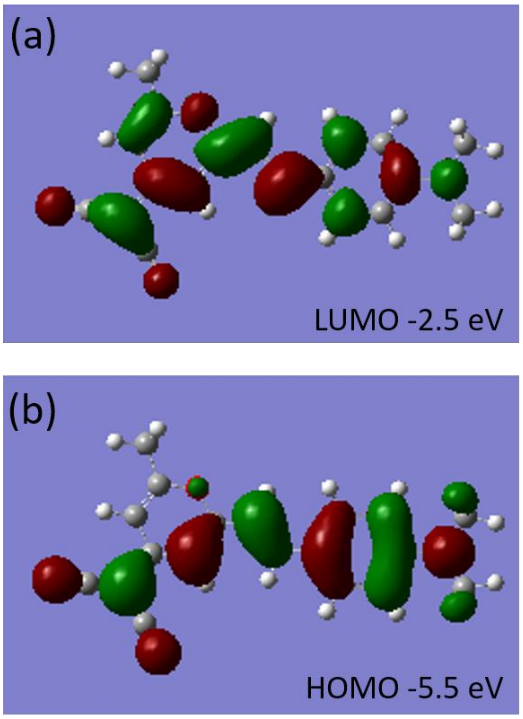

(c)

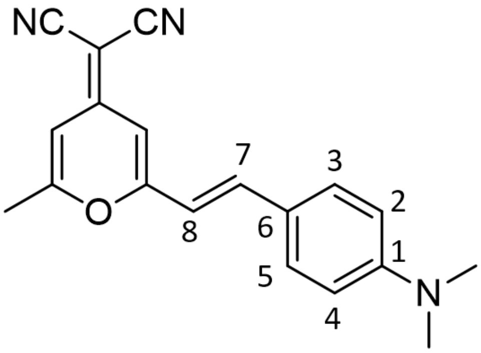

Carbon Atom Number	Natural Charge Value
1	0.21016
2	-0.26603
3	-0.13740
4	-0.25615
5	-0.14350
6	-0.13740
7	-0.10450
8	-0.27289

Figure S6. (**a**) LUMO and (**b**) HOMO electron distributions and energy levels calculated for the DCM molecule. (**c**) The calculated charge distribution (both symbol and density) among the eight carbons within styrene backbone. The calculations were performed with density-functional theory (td-B3LYP/6-311g**//b3lyp/6-31g*) using the Gaussian 03 package.

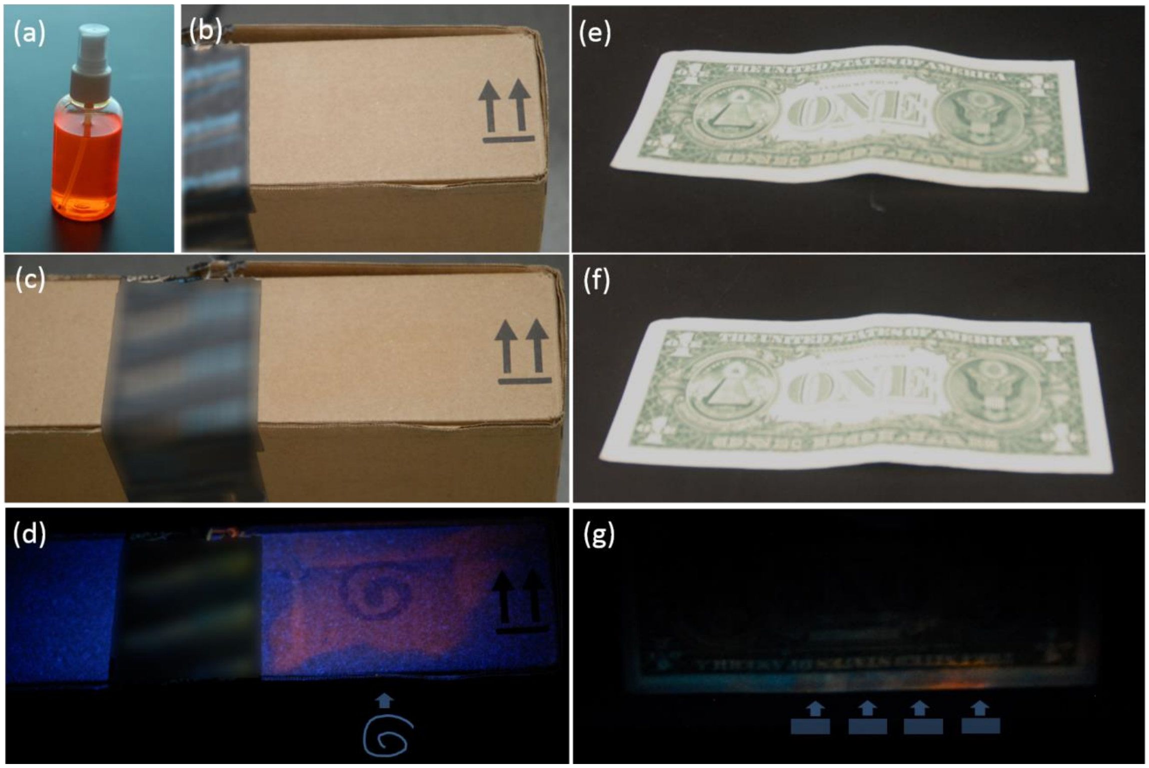

Figure S7. (a) The DCM solution (3×10^{-3} mol·L^{-1}) prepared in a 1:1 (v/v) binary solvent of acetonitrile and ethanol. (b, c, d) The photographs taken for a packing cardboard (the band name on the black tape was blurred) preloaded with ca. 100 μg RDX on the surface, the same surface after sprayed with the DCM solution, and after 2 min of UV irradiation at 254 nm the graphic pattern of the preloaded RDX is clearly visioned as dark (quenched) area in contrast to the surrounding photoluminescence of DCM. (e, f, g) The photographs taken for a dollar bill preloaded with RDX in four discrete areas along the edge (each area containing ca. 20 μg RDX), the same bill sprayed with the DCM solution, and after 2 min of UV irradiation at 254 nm the four areas preloaded with RDX are clearly visioned as dark (quenched) domains in contrast to the surrounding photoluminescence of DCM.
